# Integrated Circuit Design for Radiation-Hardened Charge-Sensitive Amplifier Survived up to 2 Mrad

**DOI:** 10.3390/s20102765

**Published:** 2020-05-12

**Authors:** Changyeop Lee, Gyuseong Cho, Troy Unruh, Seop Hur, Inyong Kwon

**Affiliations:** 1 Department of Nuclear and Quantum Engineering, Korea Advanced Institute of Science and Technology, Daejeon 34141, Korea; changyeop-lee@kaist.ac.kr (C.L.); gscho1@kaist.ac.kr (G.C.); 2 Korea Atomic Energy Research Institute, Daejeon 34057, Korea; shur@kaeri.re.kr; 3 Idaho National Laboratory, Idaho Falls, ID 83415-3531, USA; troy.unruh@inl.gov

**Keywords:** charge-sensitive amplifier, total ionizing dose, radiation effects, application-specific integrated circuit (ASIC), readout circuit, nuclear power plant, preamplifier, operational amplifier

## Abstract

According to the continuous development of metal-oxide semiconductor (MOS) fabrication technology, transistors have naturally become more radiation-tolerant through steadily decreasing gate-oxide thickness, increasing the tunneling probability between gate-oxide and channel. Unfortunately, despite this radiation-hardened property of developed transistors, the field of nuclear power plants (NPPs) requires even higher radiation hardness levels. Particularly, total ionizing dose (TID) of approximately 1 Mrad could be required for readout circuitry under severe accident conditions with 100 Mrad around a reactor in-core required. In harsh radiating environments such as NPPs, sensors such as micro-pocket-fission detectors (MPFD) would be a promising technology to be operated for detecting neutrons in reactor cores. For those sensors, readout circuits should be fundamentally placed close to sensing devices for minimizing signal interferences and white noise. Therefore, radiation hardening ability is necessary for the circuits under high radiation environments. This paper presents various integrated circuit designs for a radiation hardened charge-sensitive amplifier (CSA) by using SiGe 130 nm and Si 180 nm fabrication processes with different channel widths and transistor types of complementary metal-oxide-semiconductor (CMOS) and bipolar CMOS (BiCMOS). These circuits were tested under γ–ray environment with Cobalt-60 of high level activity: 490 kCi. The experiment results indicate amplitude degradation of 2.85%–34.3%, fall time increase of 201–1730 ns, as well as a signal-to-noise ratio (SNR) of 0.07–11.6 dB decrease with irradiation dose increase. These results can provide design guidelines for radiation hardening operational amplifiers in terms of transistor sizes and structures.

## 1. Introduction

Radiation detectors have been widely used in nuclear power plants (NPPs). For instance, measuring neutron flux in a reactor core of a NPP delivers critical information for safety operations. An up-to-date neutron detector known as a micro-pocket-fission detector (MPFD) has been introduced that can combine the operational concept of the coaxial fission chamber with radiation hardening geometry [1]. Generally, a containment building in NPPs presents a relatively high total dose and dose rate, $10^{8}–10^{9}$ krad and $10^{3}–10^{6}$ krad/h, unlike the outer space field with $10^{3}–10^{4}$ krad total dose and $10^{-4}–10^{-2}$ krad/h dose rate, respectively [2]. For this harsh environment, readout circuits should be radiation hardened and installed as close as possible near the sensor to minimize voltage degradation and signal interferences caused by long coaxial cables.

Previous work on circuits for total ionizing dose (TID) effects have been systemically conducted for the last forty years, although it has predominantly focused on the outer space field because of the many projects organized by NASA [3,4]. This research for space applications has been mainly investigated for degradation of single transistor performances such as threshold voltage shift, leakage current increase, trans-conductance reduction, and electrical noise increase caused by radiation [5,6,7,8,9,10]. Furthermore, radiation influences for unit circuits, for instance, bandgap reference circuit and bipolar CMOS (BiCMOS) amplifier, were analyzed in terms of output direct current (DC) voltage balance, spurious-free dynamic range (SFDR), etc. [11,12,13,14,15]. Similar to the developed circuits for space environments, suitable radiation hardened readout circuits for NPP conditions have become more highly required in these days.

Particularly, a preamplifier, which directly amplifies current signals from detectors to voltage signals, is used for downstream signal processing to a shaping amplifier and an analog digital converter (ADC). Thus, the circuit is a key factor determining the performance of the radiation measurement system such as energy resolution and photon detection efficiency (PDE) [16,17]. One major architecture of preamplifiers is a charge-sensitive amplifier (CSA), which consists of an operational amplifier (OP-Amp), a feedback capacitor, and a feedback resistor [18,19]. It could be considered as a front-end readout circuit in a wide range of applications using detectors, since this circuit converts collected charges from a detector into voltage pulses with excellent converting linearity despite temperature, DC bias voltage, and gain variations [20]. However, these advantages could be debased from amplitude and fall time variations as well as electrical noise increase caused by induced radiation. 

For the reason, this paper provides the radiation-hardened designs of a CSA operating in harsh radiating environments such as NPPs by using channel width optimization of CMOS and BiCMOS transistors in comparison of 130 nm SiGe and 180 nm Si fabrication technologies. These schemes are associated with electrical noise of transistors, transistor type (bipolar junction transistor (BJT) and metal-oxide semiconductor field effect transistor (MOSFET)), and gate-oxide thickness depending on fabrication technologies. In order to clear the effect of numerous control variables on the circuit level, experimental results for amplitude change, fall time variation, and electrical noise are presented within a dose rate range corresponding to NPP environments.

## 2. Total Ionizing Dose Effects on MOSFET and BJT

When radiation is injected into a metal-oxide semiconductor field effect transistor (MOSFET) and bipolar junction transistor (BJT), electron hole pairs (EHP) are formed along most of the radiation path, including the oxide layer. In the substrate, EHPs can be immediately recombined, while the holes of EHPs can be trapped in an oxide layer due to a hole-hopping-mechanism [21]. These holes result in total ionizing dose (TID) effects, which cause electrical malfunctions by triggering threshold voltage shifts, transconductance ($g_{m}$) degradation, electrical noise increase, and leakage current increase [5,6,7].

### 2.1. Threshold Voltage Shift of MOSFET

The threshold voltage shift is the result of charges trapped inside the oxide ($\Delta V_{\mathrm{OT}}$) and the interface ($\Delta V_{\mathrm{IT}}$) between silicon dioxide (${\mathrm{SiO}}_{2}$) and the channel of a MOSFET [8]. First, we have:

(1) $$\Delta V_{OT}=-\frac{q}{C_{OX}}\Delta N_{OT}=-\frac{q}{\varepsilon_{OX}}t_{OX}\Delta N_{OT}\;,$$

where *q* is elementary charge, and $C_{OX}$ is the specific capacitance of the MOS capacitor that is expressed by $C_{OX}=\varepsilon_{OX}\frac{A}{t_{ox}}$. $\Delta N_{OT}$ is the density of oxide-trapped holes per area unit, given by $\Delta N_{OT}=\int_{0}^{t_{OX}}n_{th}\left(x\right)dx$. According to this equation, $\Delta V_{OT}$ is proportional to $t_{\mathrm{OX}}^{2}$ [8,9]

Second, interface-trapped holes change the MOSFET channel charges given by:

(2) $$\Delta V_{IT}=-\frac{\Delta Q_{IT}}{C_{OX}}=\frac{\Delta Q_{IT}}{\varepsilon_{OX}}t_{OX},$$

where $\Delta Q_{IT}$ is the interface-trapped charge density per area unit, which breaks the channel charge balance, depending on the type of MOSFET.

The overall threshold voltage shift is expressed by [8]:

(3) $$\Delta V_{OV}=\Delta V_{OT}+\Delta V_{IT}\;.$$

The Equations (1)–(3) indicate that thin $t_{OX}$ (gate-oxide) with large $C_{OX}$ leads to reduced threshold voltage shift. In terms of quantum mechanics, thin gate-oxide increases the probability of quantum tunneling of electrons. The increased probability enables most of the trapped holes caused by induced radiation to be recombined with electrons [10,11]. Therefore, minimizing $t_{OX}$ helps the MOSFET device become more radiation-tolerant due to the reduced threshold voltage shift. The previous irradiation test results of threshold voltage shift show a variation from 3 mV up to 20 mV depending on the gate-oxide thickness [7,22]. Therefore, deep submicron CMOS technologies with a thin gate oxide help MOSFET devices become more robust to radiation.

In this paper, CSAs fabricated by SiGe 130 nm and 180 nm CMOS technologies with different gate oxide thickness are compared, since threshold voltage shift can be expressed by amplitude variations of CSA’s output signals.

### 2.2. Noise Analysis Based on MOSFET 

Electronic noise is classified into two mechanisms: electron velocity fluctuations corresponding to thermal noise and electron number fluctuations representing 1/f noise. Both types of electric noise are relatively increased by induced radiation into MOSFET. The noise in MOSFET is expressed by:

(4) $$\frac{d\bar{e^{2}}}{df}=S_{w}+\frac{A_{f}}{f}=\frac{4k_{B}T\Gamma }{g_{m}}+\frac{K_{f}}{C_{OX}WLf}\;,$$

where $\bar{e^{2}}$ is a variance of a voltage source represented by means of power spectral density. $\frac{4k_{B}T\Gamma }{g_{m}}$, the frequency independent term, is white noise ($S_{w}$) dominated by the channel thermal noise and consists of Boltzmann’s constant ($k_{B}$), absolute temperature (T), channel thermal noise coefficient (Γ), and channel transconductance ($g_{m}\propto \frac{W}{L}$). The term, $\frac{K_{f}}{C_{OX}WLf}$, which is known as the 1/f noise or the flicker noise, is inversely proportional to the frequency and consists of 1/f noise parameter ($K_{f}$), capacitance ($C_{OX}$) of the MOSFET, and channel width and length (W, L). According to Equation (4), it is possible to reduce the electric noise by increasing the channel width [5].

Optimizing sizes of MOSFETs would lead to a more radiation tolerant circuit when designing a radiation hardening device, such as reducing electric noise and increasing the channel width. For the comparison, CSAs have been designed with two types of the channel widths of 1 µm and 2 µm.

### 2.3. Gain Degradation of BJT

A p-type MOSFET device has radiation-hardening characteristics, because major carriers are not electrons but holes. Additionally, an npn BJT exhibiting lower 1/f noise than n-type MOSFET has been widely used for applications requiring low noise and high SFDR [15,23]. Therefore, an OP-Amp combining npn BJTs with p-type MOSFETs should be investigated regarding the radiation tolerance by replacing the n-type MOSFETs.

The base currents for the npn BJTs are composed of recombination current ($I_{B1}$) and injection current ($I_{B2}\;$) caused by the carrier transfer between base and emitter terminals [24]. First, the recombination current is expressed by:

(5) $$I_{B1}=\frac{Q_{B}}{\tau_{b}},$$

where $Q_{B}$ is total quantity of electrical charges of minority carriers inside the base region. The small minority carrier life-time ($\tau_{b}$) means that the most of the carriers have been recombined faster than large $\tau_{b}$.

Second, the injection current provoked by a hole injection from base into emitter region is given by:

(6) $$I_{B2}=\frac{qAD_{p}}{L_{p}}P_{E}\left(0\right)=\frac{qAD_{p}}{L_{p}}\frac{n_{i}{}^{2}}{N_{D}}exp\frac{V_{BE}}{V_{T}},$$

where $L_{p}$ is the diffusion length of minority carriers inside the emitter region, $D_{p}$ is the diffusion coefficient of holes, *q* is the magnitude of charges, A is the sectional area, and $P_{E}\left(0\right)$ is the concentration of the minority carriers injected into the emitter from the junction of the base region. The total base current can be obtained by summations ($I_{B}=I_{B1}+I_{B2}$). Then, the current gain ($\;\beta_{F}$) is expressed by [20]:

(7) $$\beta_{F}=\frac{I_{C}}{I_{B}}=\frac{1}{\frac{W_{B}{}^{2}}{2\tau_{b}D_{n}}+\frac{D_{p}}{D_{n}}\frac{W_{B}}{L_{p}}\frac{N_{A}}{N_{D}}},$$

where $W_{B}$ is the width of the base region, $D_{n}$ is the diffusion coefficient of electron, and the $N_{A}/N_{D}$ is the relative doping ratio of the base region and the emitter region. Previous studies have indicated that a dominant factor of the gain degradation in case of npn BJTs from Equation (7) is reduction of carrier life-time ($\tau_{b}$) due to electrons inside the base region recombined with trapped holes provoked by radiation inside the isolation oxide between the emitter and the base terminals [6].

### 2.4. Noise Analysis Based on BJT

The noise source of BJTs is composed of the thermal noise of minority carriers spreading resistance from the base to the emitter region, shot noise associated with the junction potential barrier of the base and corrector current, and 1/f noise of the base current [25]. Similar to the gain degradation mechanism of BJTs, a predominant noise source by induced radiation is 1/f noise rather than thermal and shot noise due to generation of the additional traps [26]. In our experiments, average mean-square variations of output voltages were measured from Gaussian distributions of amplitudes at the baseline of CSA output signals.

## 3. Designed Charge-Sensitive Amplifier

**Figure 1:**
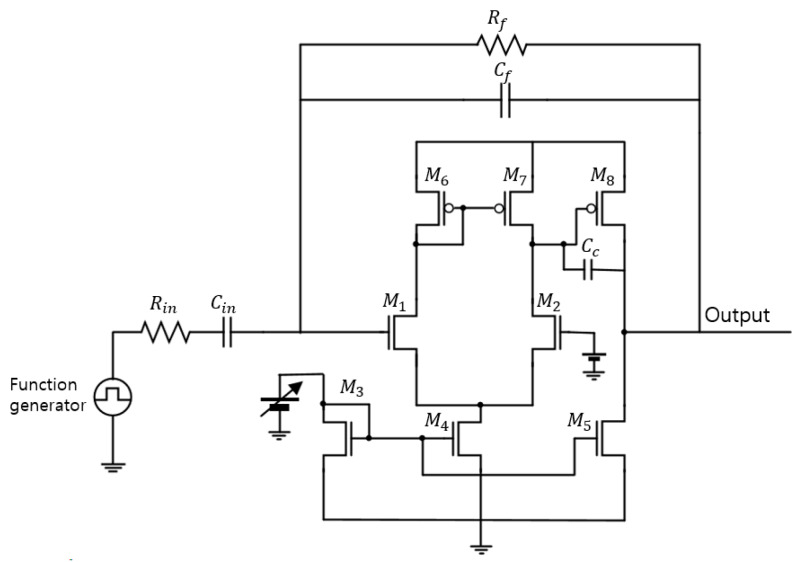
Schematic of the designed CSAs including a feedback resistor and a capacitor with the input configuration.

As illustrated in Figure 1, CSAs were designed with a two-stage operational amplifier, an external feedback resistor ($R_{f}$) and an external feedback capacitor ($C_{f}$). In the figure, $M_{1}$ and $M_{2}$ are considered as differential inputs of the first stage, and $M_{3}$-$M_{5}$ are placed as current mirrors. The $M_{6}\;$ and $M_{7}$ act as the active load and $M_{8}$ acts as a common source of the second stage.

**Figure 2:**
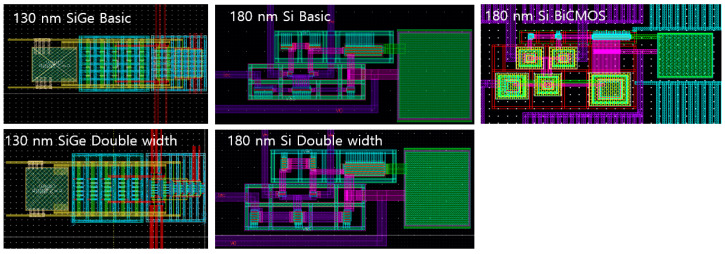
The layout of CSAs fabricated for irradiation tests.

**Figure 3:**
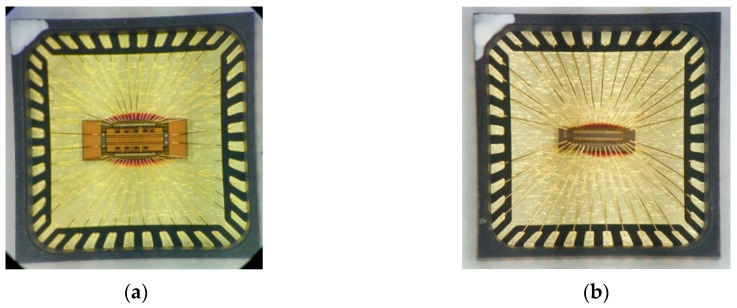
The microphotographs of packaged CSAs (**a**) of 130 nm SiGe fabrication and (**b**) of 180 nm Si fabrication.

For various samples, CSAs were designed with different variables, as shown in Table 1. In order to analyze the amplitude and the fall time variation from induced radiation, the output voltage ($V_{out}$) of the CSA should be considered by:

(8) $$V_{out}=\frac{Q_{s}}{jwC_{f}+\frac{jw}{A_{OL}\left(jw\right)}\left(C_{f}+C_{D}\right)},$$

where $\frac{1}{jwC_{D}}$ and $\frac{1}{jwC_{f}}$ are impedance of detector capacitance and a feedback capacitance depending on the frequency. The open-loop gain ($A_{OL}$) of two stage OP-Amps at low frequency is expressed by:

(9) $$A_{OL}=2g_{m2}g_{m8}\left(r_{o2}//r_{o7}\right)\left(r_{o5}//r_{o8}\right)\;,$$

where $g_{m}$ and $r_{O}$ are respectively the trans-conductance and the output resistance of MOSFET from Figure 1. Considering these equations and methods, the CSAs were designed with the similar specifications of approximately 40 dB gain and 60° phase margin, as shown in Table 1 and Figure 2. These CSAs fabricated in 130 nm SiGe and 180 nm Si were packaged, as shown in Figure 3.

## 4. Experimental Setup

**Figure 4:**
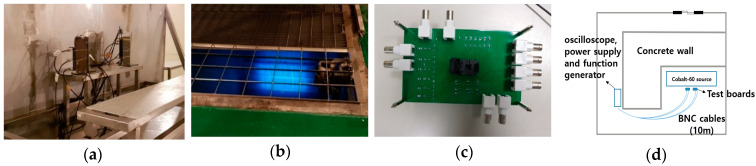
The photographs of (**a**) the experiment test setup with 10 m BNC coaxial cables, (**b**) the γ–ray irradiation source of Cobalt-60, (**c**) the test board, and (**d**) the test configuration.

The γ–ray irradiation tests were performed with Cobalt-60 of 490 kCi at Korea Atomic Energy Research Institute (KAERI). According to the standard dose rate of European Space Components Coordination (ESCC) 22900 [27], we exposed the CSAs to up to 2 Mrad with the dose rate of 104.43 krad/h. The test boards and equipment for the measurements were placed away from the irradiation room with 10 m BNC coaxial cables, as shown in Figure 4.

## 5. Experimental Results

**Figure 5:**
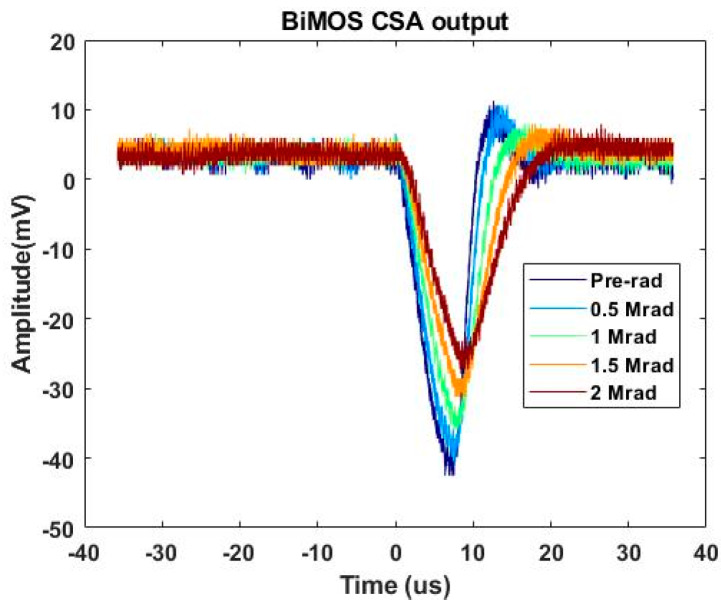
During the irradiation test with Cobalt-60 γ-ray exposure up to 2 Mrad (SiO_2_), transient signals were measured.

The input current pulse from a function generator was set to 200 nA and 3–4 µs to supply all of the CSAs. In addition, positive supply voltage of 1.8 V, gate-source voltage of current mirror of 0.6 V, and positive input voltage of 0.9 V were applied. For the entire experiments, output signals of CSAs were measured in real time using a high-end oscilloscope at 20 GHz sampling rate and 200 MHz bandwidth. Under these test environment, transient signals of BiCMOS CSA were saved, as shown in Figure 5.

### 5.1. Normalized Amplitude

**Figure 6:**
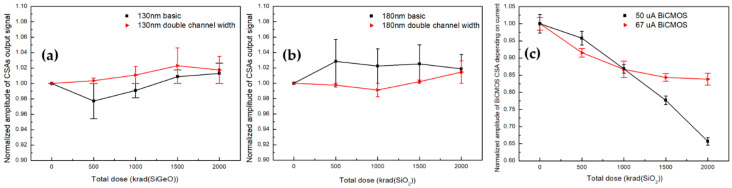
During the irradiation test with Cobalt-60 γ-ray exposure up to 2 Mrad, normalized amplitude are plotted for (**a**) 130 nm SiGe (**b**) 180 nm Si, and (**c**) BiCMOS. Measured maximum amplitudes have variations from 2.23%–34.3% depending on the designs of the CSAs with different fabrications, channel widths of metal-oxide semiconductor field effect (MOSFET), and current flows in BiCMOS.

After the irradiation tests, amplitudes of CSAs were investigated, as shown in Figure 6. In the case of the 130 nm SiGe CSA in Figure 6a, normalized maximum amplitude variations of the basic size and double channel widths are respectively shown as 3.6% and 2.23% (($Amp_{max}$-$Amp_{min}$)/$\;Amp_{pre-rad}\times 100$) during the total dose 2 Mrad (SiGeO). In addition, 180 nm Si of Figure 6b shows 2.85% and 2.32% for the basic size and the double channel widths, respectively. The maximum amplitude variations could be generated by induced γ-ray radiation, as considered with previous results [7] and Equations (8) and (9), thus this phenomenon is related to the steady decline of trans-conductance, including a threshold voltage term, and the steady increase of the output resistance [28,29]. Moreover, Figure 6a,b shows that increasing the channel width without modifying fabrication processes could deliver more radiation tolerance against amplitude variations. These results are associated with threshold voltage shifts depending on channel widths. The threshold voltage shift varies in a larger range for the basic size design compared to the doubled channel width design [30,31].

As shown in Figure 6c, CSAs based on BiCMOS with different current flows (50 μA, 67 μA) are plotted for amplitude variations. In the case of 50 μA, 34.3% degradation of amplitudes appears in comparison with 16.17% degradation of 67 μA. These sharp decreases in amplitude, unlike CMOS designs, can be explained with Equations (5) and (6), since generated traps as a result of radiation impact events inside an oxide layer can lead to base current ($I_{B}$) increase by reducing minority carrier lifetime. Furthermore, a low current flow can cause more amplitude degradation than a high current flow, because the high current has higher number of minority carriers.

### 5.2. Fall Time

**Figure 7:**
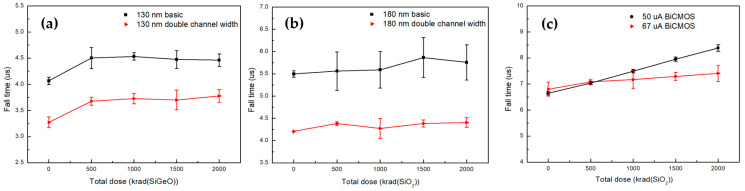
During the irradiation test with Cobalt-60 γ-ray exposure up to 2 Mrad, fall times were measured for (**a**) 130 nm SiGe (**b**) 180 nm Si, and (**c**) BiCMOS at 10% and 90% of peak voltages. The fall times show variations from 201–1730 ns depending on the CSAs. All of the CSAs appear with the increased fall time. Especially, the BiCMOS shows relatively greater slope.

Figure 7 shows that all of the CSAs have increased fall time from 201–1730 ns calculated by ($FT_{pre-rad}-FT_{\;2\;Mrad}$). As shown in Figure 7a, the 130 nm CSAs have fall time variations of 3.6 ns and 2.23 ns depending on basic and double channel widths, respectively. The 180 nm CSAs, as shown in Figure 7b, have similar fall time variations of 2.85 ns of basic and 2.32 ns of double channel widths and of devices fabricated by the 130 nm process. These fall time increases can explained by the carrier recombination of trapped holes inside the oxide layer caused by ionizing radiation, since the slew rate (SR $=\frac{C_{c}}{I_{4}}$) is determined by the current of $M_{4}$ in Figure 1.

In comparison between two CSAs of 50 μA and 67 μA current flows, as shown in Figure 7c, the fall times increase with 608 ns and 1730 ns, respectively. As with MOSFETs, the fall times of BiCMOS are clearly increased with the slew rate decrease due to the mobile carrier recombination.

### 5.3. SNR

**Figure 8:**
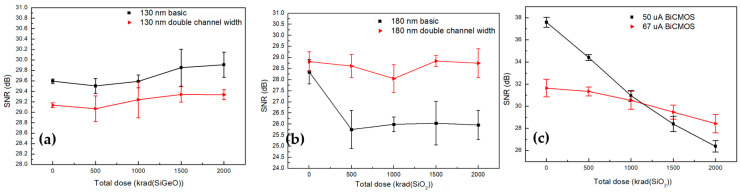
During the irradiation test with Cobalt-60 γ-ray exposure up to 2 Mrad, standard deviation of the Gaussian distribution for signal-to-noise ratios (SNRs) were measured for (**a**) 130 nm SiGe, (**b**) 180 nm Si, and (**c**) BiCMOS. In the case of the 180 nm basic design, the SNR sharply drops as 2.59 dB after 500 krad (SiO_2_) in total dose.

The changes of signal-to-noise ratios (SNR) were measured from 0.07–11.6 dB according to the total dose increase, as shown in Figure 8. The 130 nm CSAs show small changes of −0.314 and −0.202 dB depending on the basic and the double channel widths exposed to up to 2 Mrad (SiGeO) in total doses in Figure 8a. On the other hand, the SNRs for 180 nm designs show relatively higher degradations of 2.396 and 0.07 dB for the basic and the double channel widths, respectively, in Figure 8b. Especially, the basic channel width design is sharply decreased after 500 krad (${\mathrm{SiO}}_{2}$) in total dose. These results are related to noise increase corresponding to 1/f and thermal noise. Depending on Equation (4), the electrical noise of MOSFET is relatively reduced by the increased channel width. Furthermore, these noises of CSAs fabricated in 130 nm SiGe are scarcely seen, because of the increased probability of the quantum tunneling of electrons in the thinner gate oxide rather than the 180 nm CSA, which can leave fewer trapped holes inside the oxide layer; this was explained by the previous work for a single transistor [32].

In the case of BiCMOS designs, SNR reductions of 11.6 dB and 3.12 dB are shown by the current flows of 50 μA and 67 μA, as shown in Figure 8c. As mentioned in Section 2.4, 1/f noise, which is associated with the carrier number fluctuation, becomes much worse by accumulated radiation effects due to the recombination process between mobile carriers and trapped holes. For this reason, incident radiation can bring SNR degradation. Moreover, a high current flow with larger mobile carriers can be more radiation tolerant than a low current flow with fewer mobile carriers for CSAs.

## 6. Conclusions

This paper provides a comparison of different CSA designs in order to be operated in high radiation environments designed with different fabrication technologies, channel widths, and transistor types. After the irradiation tests, although fabricated CSAs survived up to 2 Mrad γ-ray with generating valid output signals, the influences of radiation showed amplitude degradation from 2.85% to 34.3%, fall time increase of 201–1730 ns, as well as SNR decrease of 0.07–11.6 dB for all CSAs, as summarized in Table 2. Note that the BiCMOS CSA with 50 uA shows the worst performance, with amplitude decrease of 34.3%, fall time increase of 1730 ns, and SNR reduction of 11.6 dB. However, the remarkable thing we came up with is that increasing current flow for the BiCMOS CSAs can notably improve the performance of the CSA. Furthermore, in comparison with CMOS and BiCMOS technologies, the CSA designs that consisted of CMOS transistors showed more radiation tolerance in terms of amplitude, fall time, and SNR. These results are primariy associated with gain degradation of BiCMOS caused by operation mechanisms and structures, unlike CMOS.

The experimental results in this paper indicate that increasing channel width and current flow and using deep−submicron MOSFETs help CSAs become more radiation tolerant with excellent converting linearity operating in harsh radiation environments such as NPPs. The data may be useful for circuit designers for applications requiring radiation hardened ability.

## Figures and Tables

**Table 1:** Designed variable OP-Amps with different sizes versus properties of gain, gain-bandwidth product and phase margin.

Variable CSA	130 nm SiGe CMOS	130 nm SiGe CMOS	180 nm Si CMOS	180 nm Si CMOS	180 nm Si BiCMOS	180 nm Si BiCOMS
	Basic	Double Width	Basic	Double Width	Tail Current 50 μA	Tail Current 67 μA
$M_{1}$ and $M_{2}$ W/L (µm)	1/0.13	2/0.13	1/0.18	2/0.18	2/2	2/2
$M_{4}$ W/L (µm)	4/0.13	8/0.13	4/0.18	8/0.18	2/2	2/2
$M_{6}$ and $M_{7}$ W/L (µm)	3/0.13	6/0.13	3/0.18	6/0.18	10/0.18	10/0.18
Gate oxide thickness (nm)	2	2	3.6	3.6	3.7	3.7
Shallow Trench Isolation (nm)	400	400	350	350	320	320
Gain (dB)	41.9	42.3	39.37	38.83	62.43	62.43
Gain Bandwidth Product (MHz)	316.2	377.3	202.25	289.5	115.24	115.24
Phase Margin (˚)	56.46	57.6	58.72	57.5	44.2	44.2

**Table 2:** Summary for irradiation test results with amplitude, fall time, and SNR.

Variable CSA	130 nm SiGe CMOS	130 nm SiGe CMOS	180 nm Si CMOS	180 nm Si CMOS	180 nm Si BiCMOS	180 nm Si BiCMOS
	Basic	Double Width	Basic	Double Width	Tail Current 50 μA	Tail Current 67 μA
Amplitude variation (%) ($Amp_{max}-Amp_{\;min}$)	3.6	2.23	2.85	2.32	34.3	16.17
Fall time variation (ns) ($FT_{pre-rad}-FT_{\;2000\;krad}$)	−398	−504	−260	−210	−1730	−608
SNR (dB) ($SNR_{pre-rad}-SNR_{2000\;krad}$)	−0.314	−0.202	2.396	0.07	11.6	3.12
